# Measuring American adults’ perceptions about human existence: A cross-sectional study

**DOI:** 10.1017/S1478951525100497

**Published:** 2025-08-22

**Authors:** Megan Rose Carr LaPorte, Linda Emanuel, Sheldon Solomon, Carolinne Viana Poffo, Isha Joshi, Yingwei Yao, Diana J Wilkie

**Affiliations:** 1 Department of Medicine, Cambridge Health Alliance, Cambridge, MA, USA; 2 Harvard Medical School, Boston, MA, USA; 3 Mongan Institute, Harvard University, Boston, MA, USA; 4 Supportive Oncology, Robert H. Lurie Comprehensive Cancer Center, Northwestern Medical Group, Feinberg School of Medicine, Northwestern University, Chicago, IL, USA; 5 Department of Psychology, Skidmore College, Saratoga Springs, New York, IL, USA; 6 Department of Biobehavioral Nursing Science, College of Nursing, University of Florida, Gainesville, FL, USA

**Keywords:** Existential maturation, terror management theory, explicit death anxiety, implicit death anxiety, adult attachment

## Abstract

**Objectives:**

Awareness of death shapes our existence; it prompts both distress and a maturation process called existential maturation. Presently, direct quantitative measures of existential maturation are unavailable to study treatments for existential distress that enhance psychological well-being. We examined the effect of a mortality salience stimulus on implicit death thoughts over time. We also examined the associations among existing measures of constructs conceptualized as relevant to an eventual measure of existential maturation in a representative sample.

**Methods:**

A cross-sectional Qualtrics panel of 1,000 adults, representative of the United States' urban and rural populations, completed a 20-minute survey. The self-report Human Existence survey included an embedded mortality salience stimulus (Death Anxiety Beliefs and Behaviors Scale) and valid, reliable measures of implicit death-thought accessibility (DTA), existential isolation, existential distress, flourishing, transcendence, attachment, connections, peace, and other related constructs.

**Results:**

The DTA measure did not replicate previous research on mortality salience. We found significant positive correlations between existential isolation and existential distress, and between flourishing and transcendence. However, correlations of death anxiety with isolation, flourishing, and transcendence were surprisingly low. In multivariate analysis, avoidant attachment was negatively associated with existential isolation and distress; death anxiety was positively associated with anxious/ambivalent attachment. Transcendence was negatively associated with avoidant attachment and positively associated with being at peace and connections. Flourishing was positively associated with being at peace and connections.

**Significance of results:**

An ineffective death reminder or the DTA online format may have affected DTA results. Striking relationships between attachment style and EM indicators confirm they are interrelated. Measures for existential maturation and related phenomena still lack implicit measures to assess nonconscious components.

## Introduction

“… it is our knowledge that we have to die that makes us human …”

Alexander Smith (Smith 1863)

Awareness of death fundamentally shapes our existence, driving a lifelong process in which individuals, in varied ways, integrate the inevitability of death into their being. The awareness of death not only prompts existential terror but also motivates searches for purpose, significance, and connection. This process of existential maturation has been described clinically, socio-culturally, and theoretically. Our study was an initial step to develop a measure of existential maturation.

To do so, we built on terror management theory research that has established the following: 1) High self-esteem reduces self-reported anxiety and physiological arousal in response to psychological and physical threats. 2) Reminding people of their own death (*mortality salience* – e.g., by asking people to reflect on their death, interviewing people in front of a funeral home, or subliminal exposure to the word “dead” or “death”) instigates cultural worldview defense and self-esteem striving. 3) Threats to cherished cultural beliefs, self-esteem, or significant personal relationships make death thoughts come more readily to mind (*death-thought accessibility*; DTA). Additional research (Pyszczynski et al., 1999) has established that death thoughts instigate distinct defensive reactions.

Existential maturation is the flip side of existential terror. Encounters with mortality, such as the death of a significant other, being diagnosed with a terminal illness, wars, environmental catastrophes, economic and political instabilities, and pandemics, affect us deeply. The COVID-19 pandemic was a significant case in point for the global population (Pyszczynski et al., 2021; Vacchiano et al., 2023). Responses to such mortality reflect a person’s existential maturation. Existential maturation describes a developmental process. A healthy relationship to mortality is one that does not rely on maladaptive defenses (Emanuel 2021). Existential maturity does not spare people the pain and grief that come with dying and loss, but it develops psychological and relational resources to process these matters. The journey toward existential maturation is nonlinear (Emanuel 2023) and is variably attained (Emanuel 2021). Those who work with dying patients can often recognize when patients are at peace with death and when they are not, and how they oscillate between these states (Brenner et al., 2021).

Emanuel’s psychophysiological model proposes that existential maturation arises from processing mortality-salient events within a containing relational context, leading to an integrated understanding and resilience (Emanuel 2023). Within this model, attachment is critical for existential maturation, and untreated trauma significantly hinders the development of existential maturation (Emanuel and Brody, 2022). It follows that the development of existential maturation can be aided by treatments such as psychodynamic/analytic work, existential therapy (Emanuel et al., 2021; Yalom, 1980), or Dignity Therapy (Chochinov et al., 2005; Emanuel and Scandrett, 2010).

There are several relevant measures, including those for death anxiety; however, a direct quantitative measure of existential maturation has been elusive, limiting the empirical study of this important phenomenon. A crucial part of the challenge stems from the nature of the phenomenon. Death anxiety and existential maturation are both nonconscious/implicit as well as conscious/explicit. Nonconscious phenomena are not simply correlated with conscious phenomena. For instance, previous research (Greenberg et al., 1995) has found that people who reported the lowest levels of explicit death anxiety responded most strongly (made choices that eschewed death) to a mortality salience induction (reminder of death). Furthermore, many of the relevant scales are old, so normative data may be out of date. Obtaining a large representative sample is, therefore, important. Finally, it is relevant to study possible differences between rural and urban populations. We consequently conducted a panel study in a general population to examine the effect of a mortality salience stimulus on implicit death thoughts over time, to assess nonconscious death anxiety, and to examine the associations among measures of constructs that are relevant to an eventual measure of existential maturation.

## Methods

### Study design

**Figure 1.**
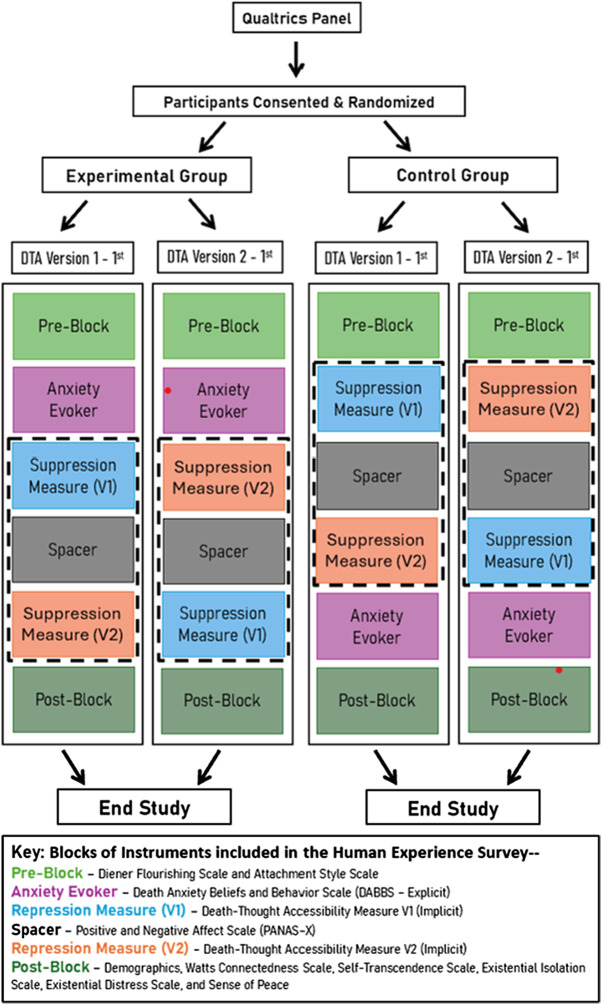
Study design diagram showing the four groups into which participants were randomized.

In a single-session survey with randomization to four study groups (Figure 1), we examined the effect of mortality salience on measurable psychosocial outcomes while also testing the order effects of the implicit death anxiety measure. This design allowed us to evaluate the associations between existential maturation theoretical constructs (e.g., well-being, attachment, connectedness, death anxiety, and existential issues). The University of Florida’s Institutional Review Board evaluated the study as exempt.

### Sample

We used a Qualtrics panel to obtain a representative sample of 1,000 U.S. residents based on age, gender/sex, race, and rural/urban residence for our survey. Inclusion criteria required participants to be aged 18 years or older and able to read English.

### Procedures

The participants were randomly assigned to groups, and the order of the instruments varied by group, as listed in Figure 1. The order of instruments was designed to test the viability of a measure of implicit (i.e., nonconscious) death anxiety based on the placement of a death-thought-accessibility measure. The participants accessed the 20–30-minute Human Existence survey on their own devices via a Qualtrics link. All data were anonymous to the investigators, as the participants’ contact information was known only to Qualtrics operators, who did not disclose identifiers and provided participant reimbursement.

### Instruments

**Figure 2.**
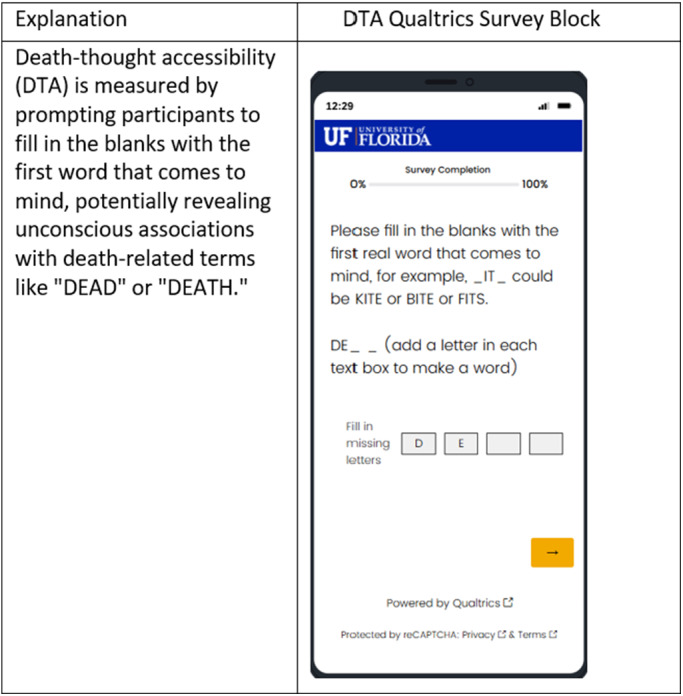
Participant view of Qualtrics survey showing word-stem completion task.

The survey included valid and reliable measures, as listed in Figure 1. We used a mortality reminder intervention (mortality salience) either before or after the participants completed the two word-stem sets, which were separated by other measures to allow 3–5 minutes between the first and second sets, as successfully used by others (Florian and Mikulincer, 1998). We counterbalanced the presentation order of the two word-stem sets, disabled the autofill function on the web-based survey, and allowed participants to enter only one letter at a time (Figure 2).

**Flourishing**. The Diener Flourishing Scale (Diener et al., 2010) is an 8-item measure of the participant’s overall perception of success regarding self-esteem, purpose, optimism, and relationships. This measure has been validated in many samples (Schotanus-Dijkstra et al., 2016), and confirmatory factor analysis revealed a good fit with a one-factor model and good internal consistency (α = .86).

**Adult Attachment Style** was measured by responses to Hazan and Shaver’s (1987) 3-item questionnaire focused on secure, anxious/ambivalent, and avoidant attachment styles. The scale has been used in large national samples and cross-cultural studies with moderate reliability, which may reflect situational variation (Russ et al., 2024; Sagone et al., 2023).

**Explicit Death Anxiety** was measured by the Death Anxiety Beliefs and Behaviors Scale (DABBS)(Menzies et al., 2022). It was also used as a death salience intervention. This recently developed 18-item scale has demonstrated good construct validity, criterion validity, internal consistency, and test-retest reliability (Menzies et al., 2022). Moreover, the DABBS effectively distinguished participants with clinically important death anxiety and distress from those without, demonstrating excellent discriminant validity.

The **Implicit Death Anxiety Death-Thought Accessibility Measure** is a nonconscious measure selected as a potential tool for assessing the implicit components of existential maturation. The death-thought accessibility (DTA) tool (Hayes et al., 2010) was chosen for this purpose. A description of the DTA measures and a meta-analysis of DTA research are available (Steinman and Updegraff, 2015). In brief, the DTA is based on the number of incomplete word stems that can be completed in death-related ways (e.g., C O F F _ _ could be COFFEE or COFFIN; G R _ V E could be GROVE or GRAVE). In this project, we used two sets (DTA1 and DTA2) of 25-word stems each, where 4 in each set could be completed with either neutral or death-related words. Park and Pyszczynski, 2019) in a prior study, demonstrated that meditation and mindfulness interventions influence DTA in a predictable fashion. DTA is typically suppressed (participants complete the stems with fewer death-related words) immediately after a death reminder (mortality salience induction), and the number increases over time thereafter (Arndt et al., 1997; Greenberg et al., 1994). However, DTA was not suppressed (i.e., the score was higher because more death-related words were generated) immediately after a mortality salience induction for meditation-trained participants. Previous authors (Park and Pyszczynski, 2019) interpreted this as an indication of a less defensive reaction to mortality, which we would view as an indication of existential maturation.

**Positive and Negative Affect** was assessed using the PANAS-X, which consists of 60 items (Watson et al., 1988). The scales are highly internally consistent, generally uncorrelated, and there is ample evidence supporting the convergent and discriminant validity of the scales. In this study, the PANAS-X allowed approximately 3–5 minutes between the two DTA measures.

**Socio-demographic factors**, including age, gender, race, ethnicity, income, medical history, rural identity, and the number of immediate family deaths, were collected as composite demographic data for the purpose of analyzing the baseline characteristics predictive of instrument order effects. For the rural identity construct, participants responded to each of the six items measured on a scale from 0 (not at all) to 6 (extremely), focusing on belonging to or being from a rural community rather than being a “city” person. Higher scores indicate greater rural identity (Krok-Schoen JL, P-WA et al., 2015). Additioanly, participants responded to the Adverse Childhood Experience Questionnaire (ACE), a well-known measure of the adverse experiences of childhood that have been associated with adult mental and physical illnesses (Felitti et al. 1998).

The **Watts Connectedness Scale** (Watts et al., 2022) measures a general feeling of connection that is associated with psychological well-being. The psychometrically validated scale consists of 19-items measuring connectedness via felt-connection to self, others, and the world (Watts et al., 2022).

**Self-Transcendence Scale** (STS) is a one-dimensional scale with 15 items focused on introspective activities, outward involvement with others, and temporally living in the present or holding perspectives of the past and future that enhance the present (Reed 1986). The scale was designed to assess an expanded sense of self and has been validated in a wide variety of samples.

The 6-item **Existential Isolation Scale** (EIS) measures the feeling of being alone in one’s own internal experience or feeling as though no one else understands and shares it (independent of interpersonal loneliness) (Pinel et al., 2017). This scale shows good internal consistency (α = .83) (Pinel et al., 2017).

The **Existential Distress Scale** (Version 2) has 10-items measured on a 5-point scale, ranging from “not distressed” to “unbearably distressed,” to indicate how distressed participants felt about being alone, having an empty or meaningless life, and being a burden to others (Krause et al., 2015). The scale has sound psychometric properties that have been validated in clinical and nonclinical samples.

**At Peace** is a single item (Steinhauser et al., 2006) from the Quality of Life at the End-of-Life Measure (QUAL-E) (Steinhauser et al., 2004) that measures the individual’s general wellbeing. The item is measured on a scale of 1 (not at all) to 5 (completely). In clinical samples, the “at peace” item showed a small positive relationship with age (r = 0.24) but no significant associations with other demographic variables (Steinhauser et al., 2006).

### Data analysis

Data management and preliminary data analysis procedures were conducted using the statistical software R. Descriptive statistics, including mean, standard deviation, frequency, and percentage, were obtained. Bivariate relationships were examined using correlation, t-test, and ANOVA. Theoretically driven regression analyses were performed to examine the association between measures. Specifically, in exploratory analyses guided in part by Emanuel’s (2023) model of existential maturation, we designated the Existential Isolation Scale (EIS), Existential Distress Scale (EDS), Death Anxiety Beliefs and Behaviors Scales (DABBS), Flourishing, and Self-Transcendence Scale (STS) as outcome variables. More specifically, we viewed EIS/EDS/DABBS as measuring a lack of existential maturation and Flourishing/STS as measuring the presence thereof. Additionally, because theory and research (Pinel et al., 2017) have established that EIS and EDS represent overlapping but conceptually distinct psychological components, we predicted that these scales would be modestly correlated, both absolutely and relative to the higher correlation expected between Flourishing and STS, given that both are purportedly single-factor assessments of superordinate psychological well-being. Other measures, namely adult attachment style, PANAS-X, WCS, and At Peace, were considered as predictors (potentially mediated by attachment style). Statistical significance was set at a two-sided alpha of 0.05.

## Results

### Demographics

Table 1 presents demographic information about the sample. We generally achieved the recruitment quota distributions, except for participants who reported their race as Asian or Pacific Islander (57% of quota), American Indian/Alaskan Native or Other (35% of quota), or Hispanic ethnicity (79% of quota). A slightly larger proportion of rural residents (115% of quota) participated. More than a third reported their family income as very difficult or difficult to live on, another third were getting by, and a third were comfortable with their family income. On average, rural identity was low, with a mean of 13.3 ± 8.6 (possible score 0–30). Participants reported an average of 5.0 ± 5.0 deaths of immediate family members. The average Adverse Childhood Experiences score was 2.1 ± 2.5.

**Table 1.** Demographic characteristics

Continuous Variables		Mean ± SD
Age		49.1 ± 18.0
Rural Identity*		13.3 ± 8.6
Death in immediate family (member)		5.0 ± 5.0
Adverse Childhood Experiences (ACE)		2.1 ± 2.5

Categorical Variables	Category	N (%)
Gender	Female	509 (50.9)
	Male	483 (48.3)
	Trans	5 (0.5)
	Other	3 (0.3)
Education	< 8 yrs	11 (1.1)
	8 – 11 yrs	43 (4.3)
	HS/GED	268 (26.8)
	Vocational	61 (6.1)
	Some college	267 (26.7)
	College	262 (26.2)
	Postgraduate	88 (8.8)
Income	≤$9,999	118 (11.8)
	$10,000 – $19,999	105 (10.5)
	$20,000 – $34,999	196 (19.6)
	$35,000 – $49,999	157 (15.7)
	$50,000 – $74,999	184 (18.4)
	$75,000 – $99,999	95 (9.5)
	≥ $100,000	113 (11.3)
	Unknown	32 (3.2)
Household Income	Very difficult	167 (16.7)
	Difficult	184 (18.4)
	Getting by	325 (32.5)
	Comfortably	284 (28.4)
	Unknown	40 (4.0)
Ethnicity	Not Hispanic	853 (85.3)
	Hispanic	142 (14.2)
	Unknown	5 (0.5)
Race	American Indian	21 (2.1)
	Asian	34 (3.4)
	Black	125 (12.5)
	Mixed	35 (3.5)
	Native Hawaiian/PI	6 (0.6)
	White	749 (74.9)
	Unknown	30 (3.0)
Religion	Buddhist	12 (1.2)
	Christian	590 (59.0)
	Hindu	4 (0.4)
	Jewish	40 (4.0)
	Muslim	22 (2.2)
	Other religion	51 (5.1)
	Spiritual not religious	66 (6.6)
	No religion	179 (17.9)
	Prefer not to answer	36 (3.6)
Rural Residence	No	771 (77.1)
	Yes	229 (22.9)

Note:

* Rural Identity = Rural Identity Scale (0–30 possible, without the city item, alpha = 0.92). Quotas specified for the panel included: **age** 18–34 years (30%), 35–54 years (32%), 55 + years (38%); **gender** male (48%), female (52%), non-binary (natural fallout); **race** White (75%), Black/African American (13%), Asian or Pacific Islander (6%), American Indian/Alaskan Native or Other (6%); Hispanic ethnicity: Hispanic (18%), non-Hispanic (82%); and **residence** rural (20%), urban (80%).

### Mortality salience manipulation: Implicit death anxiety (DTA)

As displayed in Table 2, the DTA, as measured in this study, did not replicate previous findings (Study 3) (Park and Pyszczynski, 2019) – a DTA score lower immediately after a death reminder (DABBS) that then increased over time. Replication of the finding would have enabled us to infer that high DTA (more death words on the word-completion task) immediately following a mortality salience induction reflects low implicit death anxiety (or death acceptance). This would have been similar to the overcoming of typical suppression in response to death reminders that Park and Pyszczynski (2019) found in response to meditation and mindfulness. Descriptive statistics for the mortality salience manipulation groups appear in the supplemental materials, Tables 1 and 2.

**Table 2.** Counterbalanced death-thought accessibility (DTA) scores for mortality salience and control groups pre and post-PANAS-X spacer (3–5 min delay between DTA measures)

		Group	
DTA Order		Mortality Salience	Control
**DTA1 first**	N	249	240
	DTA1 pre PANAS-X	1.01 (0.79)	0.77 (0.75)
	DTA1 post PANAS-X	1.18 (0.88)	1.17 (0.85)
	DTA1 change	0.17 (1.09)	0.40 (0.98)
**DTA2 first**	N	246	265
	DTA2 pre PANAS-X	1.12 (0.89)	1.08 (0.77)
	DTA2 post PANAS-X	0.82 (0.77)	0.78 (0.75)
	DTA2 change	−0.30 (1.07)	−0.30 (1.08)

Note: The DTA change score is the score of DTA administered after PANAS-X minus the score of DTA administered before PANAS-X. The DTA1 (version 1) scores increased for both the mortality salience and control groups, which does not indicate suppression as hypothesized. The DTA2 (version 2) scores decreased for both the mortality salience and control groups, which does not indicate suppression as hypothesized.

### Descriptives: Exploration of existential maturation outcomes and predictors

Descriptive statistics and normative/comparative data for the outcome and predictor variables are presented in Table 3. The variables in the present sample were generally in the same range as existing normative and comparative data.

**Table 3.** Descriptive statistics, normative/comparative data for outcomes and predictors

Scale	Details	Current Sample	Normative/Prior Published Data
**Outcomes**			
**EIS**	Total Score Mean (SD)	2.8 (1.0)	3.74 (1.01)
**EDS**	Total Score Mean (SD)	12.9 (12.0)	7.14 (7.52)
**DABBS**	Total Score Mean (SD)	50.1 (15.2)	52.42 (13.05)
**STS**	Total Score Mean (SD)	45.7 (9.7)	43.6 (7.2)
**Flourishing**	Total Score Mean (SD)	44.2 (9.2)	43.8 (8.4)
**Predictors**			
**Attachment style**	I am somewhat uncomfortable being close to others. AVOIDANT	348, 34.8%	25%
	I find it relatively easy to get close to others… SECURE	571, 57.1%	55 − 60%
	I find that others are reluctant to get as close as I would like… ANXIOUS/AMBIVALENT	81, 8.1%	15 − 20%
**PANAS-X**	Positive mood Mean (SD)	29.6 (8.9)	29 (8.0)
	Negative mood Mean (SD)	19.1 (9.6)	15.8 (5.9)
**WCS**	TOTAL Score Mean (SD)	5.8 (2.0)	Not available
	Subscale: Mean (SD):		Not available
	Connections To Self	6.4 (2.3)	
	Connections To Others	5.2 (1.2)	Not available
	Connections To World	5.8 (2.6)	Not available
**At peace**	Total Score Mean (SD)	3.5 (1.3)	3.7 (1.2)

Key: EIS = Existential Isolation Scale; EDS **=** Existential Distress Scale; DABBS = Death Anxiety Beliefs and Behaviors Scale; STS = Self-Transcendence Scale; Flourishing = Diener Flourishing Scale; ACE = Adverse Childhood Experiences; PANAS-X = Positive and Negative Affect Schedule – Expanded Form; WCS = Watts Connectedness Scale; At Peace = item from the Quality of Life at the End-of-Life Measure (QUAL-E).

### Bivariate relationships

The correlations between the outcome variables are presented in Table 4. As expected, although there were significant positive correlations between EIS and EDS, and between Flourishing and STS, the strength of the correlation was much lower between EIS and EDS (r = .29) than between Flourishing and STS (r = .65). Moreover, as expected, the EIS and EDS were moderately and negatively correlated with Flourishing and STS.

**Table 4.** Correlation between outcomes: EIS, EDS, flourishing, STS, and DABBS

	EIS	EDS	STS	Flourishing
EDS	0.29			
STS	−0.48	−0.40		
Flourishing	−0.40	−0.36	0.65	
DABBS	0.03	0.42	−0.12	−0.08

Note: all correlations were statistically significant (*p* < .05), except for the correlation between EIS and DABBS.

Key: EIS = Existential Isolation Scale; EDS **=** Existential Distress Scale; STS = Self-Transcendence Scale; Flourishing = Diener Flourishing Scale; DABBS = Death Anxiety Beliefs and Behaviors Scale.

Interestingly, the correlations between the DABBS measure of explicit death anxiety and all of the other outcome variables, except existential distress (EDS), were surprisingly low (Table 4). Except for the correlation between DABBS and EIS, all correlations were statistically significant.

All predictor variables, except the number of family deaths, were statistically significantly associated with the five outcome variables (Table 5). Most of the correlation coefficients indicate weak linear relationships, some positive and others negative. Exceptions were the strong positive correlations between STS and the WCS and At Peace predictors, as well as the moderate positive correlations between positive mood and both STS and Flourishing, EDS and negative mood, and Flourishing and WCS and At Peace. Moderate negative correlations were observed between EIS and WCS and At Peace, and EDS and Age and At Peace. ACE was weakly correlated with all outcomes with expected directionality (negative for EIS, EDS, DABBS, and positive for Flourishing and STS).

**Table 5.** Bivariate correlations between each outcome variable and the predictors

	Outcomes				
Predictor Variables	EIS	EDS	DABBS	STS	Flourishing
Age	−0.18	−0.43	−0.22	0.19	0.13
Rural Identity	−0.09	0.13	0.09	0.22	0.18
Immediate Family Death	0.03	−0.03	0.00	−0.06	−0.04
ACE	0.22	0.32	0.12	−0.17	−0.17
PANAS-X Positive Mood	−0.27	−0.07	0.07	0.52	0.48
PANAS-X Negative Mood	0.22	0.60	0.38	−0.34	−0.37
WCS	−0.42	−0.30	−0.09	0.72	0.56
At Peace	−0.40	−0.43	−0.19	0.65	0.53

Note: all correlations were statistically significant at p < .05, except Immediate Family Death, which was not significant for any of the outcomes.

Key: EIS = Existential Isolation Scale; EDS **=** Existential Distress Scale; STS = Self-Transcendence Scale; Flourishing = Diener Flourishing Scale; DABBS = Death Anxiety Beliefs and Behaviors Scale; Rural Identity = Rural Identity Scale; ACE = Adverse Childhood Experiences; PANAS-X = Positive and Negative Affect Schedule – Expanded Form; WCS = Watts Connectedness Scale; At Peace = item from the Quality of Life at the End-of-Life Measure (QUAL-E).

Secure adult attachment style was significantly lower than the avoidant and anxious/ambivalent attachment styles for EIS, EDS, and DABBS outcomes, and significantly higher for STS and Flourishing (Table 6). None of the outcomes differed significantly by gender, except for the EDS; males reported significantly higher scores than females (Table 6).

**Table 6.** Differences in outcomes by categorical predictors

	Outcomes									
	EIS		EDS		DABBS		STS		Flourishing	
Categorical Predictors	M (SD)	*p*	M (SD)	*p*	M (SD)	*p*	M (SD)	*p*	M (SD)	*p*
Attachment: Avoidant	3.2 (1.0)	<.001	17.0 (12.0)	<.001	51.8 (15.0)	<.001	41.6 (10.2)	<.001	40.6 (9.6)	<.001
Attachment: Secure	2.5 (1.0)		10.0 (11.2)		48.4 (15.0)		48.8 (8.1)		46.7 (7.7)	
Attachment: Anxious/ Ambivalent	3.1 (0.8)		16.0 (11.3)		55.1 (15.6)		42.1 (10.6)		42.2 (11.1)	

Gender	M (SD)	p	M (SD)	p	M (SD)	p	M (SD)	p	M (SD)	p
Female	2.7 (1.1)	.10	11.9 (12.1)	.01	50.4 (14.9)	.64	45.6 (10.2)	.50	44.1 (9.1)	.54
Male	2.8 (1.0)		13.8 (11.7)		49.9 (15.5)		46.0 (9.3)		44.4 (9.2)	

Key: EIS = Existential Isolation Scale; EDS **=** Existential Distress Scale; DABBS = Death Anxiety Beliefs and Behaviors Scale; STS = Self-Transcendence Scale; Flourishing = Diener Flourishing Scale; Attachment = Adult Attachment Style.

### Multivariate relationships

Examination of the associations between each of the outcomes and theorized correlates provides additional insights about concepts relevant to existential maturation. The five analytic models included, as predictors, gender, four correlates (At Peace, ACE, WCS, and Adult Attachment), as well as the interactions between adult attachment and the other three of those four correlates (Table 7).

**Table 7.** Regression modeling of outcomes: existential distress, existential distress, explicit death anxiety, flourishing, self-transcendence

a. Outcome: Existential Isolation					
Predictor		Estimate	Std Err	*t*	*p*
Gender = Male		0.168	0.057	2.938	**.003**
Attachment = Avoidant (Secure ref)		0.354	0.112	3.156	**.002**
Attachment = Anxious/Ambivalent (Secure ref)		0.402	0.221	1.821	.07
At Peace	Secure Attachment	−0.150	0.035	−4.260	**<.001**
	Avoidant Attachment	−0.188	0.044	−4.300	**<.001**
	Anxious/Ambivalent	−0.213	0.087	−2.463	**.01**
ACE > 0	Secure Attachment	0.266	0.075	3.553	**<.001**
	Avoidant Attachment	0.205	0.106	1.933	.05
	Anxious/Ambivalent	−0.056	0.235	−0.239	.81
WCS	Secure Attachment	−0.188	0.026	−7.245	**<.001**
	Avoidant Attachment	−0.117	0.032	−3.653	**<.001**
	Anxious/Ambivalent	−0.091	0.065	−1.405	.16

b. Outcome: Existential Distress					
Predictor		Estimate	Std Err	t	*p*
Gender = Male		3.215	0.668	4.813	**<.001**
Attachment = Avoidant (Secure ref)		3.118	1.315	2.371	**.02**
Attachment = Anxious/Ambivalent (Secure ref)		−1.660	2.583	−0.643	.52
At Peace	Secure Attachment	−3.041	0.414	−7.353	**<.001**
	Avoidant Attachment	−3.795	0.513	−7.400	**<.001**
	Anxious/Ambivalent	−1.067	1.014	−1.052	.29
ACE > 0	Secure Attachment	3.363	0.878	3.831	**<.001**
	Avoidant Attachment	3.312	1.243	2.664	**.01**
	Anxious/Ambivalent	10.888	2.749	3.961	**<.001**
WCS	Secure Attachment	−1.005	0.304	−3.299	**.001**
	Avoidant Attachment	0.138	0.375	0.368	.71
	Anxious/Ambivalent	−0.458	0.757	−0.605	.55

c. Outcome: Explicit Death Anxiety (DABBS)					
Predictor		Estimate	Std Err	t	*p*
Gender = Male		−0.015	0.951	−0.016	.99
Attachment = Avoidant (Secure ref)		2.621	1.871	1.401	.16
Attachment = Anxious/Ambivalent (Secure ref)		8.309	3.676	2.260	**.02**
At Peace	Secure Attachment	−3.380	0.588	−5.744	**<.001**
	Avoidant Attachment	−1.204	0.730	−1.650	.10
	Anxious/Ambivalent	2.596	1.443	1.799	.07
ACE > 0	Secure Attachment	1.818	1.249	1.455	.15
	Avoidant Attachment	1.674	1.769	0.947	.34
	Anxious/Ambivalent	4.583	3.911	1.172	.24
WCS	Secure Attachment	−0.188	0.433	−0.433	.67
	Avoidant Attachment	0.366	0.534	0.686	.49
	Anxious/Ambivalent	1.040	1.077	0.966	.33

d. Outcome: Self-Transcendence					
Predictor		Estimate	Std Err	t	*p*
Gender = Male		−0.333	0.384	−0.866	.39
Attachment = Avoidant (Secure ref)		−1.804	0.756	−2.385	**.02**
Attachment = Anxious/Ambivalent (Secure ref)		0.094	1.486	0.063	.95
At Peace	Secure Attachment	2.589	0.238	10.884	**<.001**
	Avoidant Attachment	2.947	0.295	9.988	**<.001**
	Anxious/Ambivalent	2.856	0.583	4.894	**<.001**
ACE > 0	Secure Attachment	0.111	0.505	0.219	.83
	Avoidant Attachment	0.517	0.715	0.723	.47
	Anxious/Ambivalent	−0.924	1.581	−0.584	.56
WCS	Secure Attachment	2.790	0.175	15.931	**<.001**
	Avoidant Attachment	2.932	0.216	13.585	**<.001**
	Anxious/Ambivalent	2.872	0.435	6.598	**<.001**

e. Outcome: Flourishing					
Predictor		Estimate	Std Err	t	*p*
Gender = Male		−0.268	0.454	−0.590	.56
Attachment = Avoidant (Secure ref)		−1.308	0.895	−1.463	.14
Attachment = Anxious/Ambivalent (Secure ref)		1.762	1.757	1.003	.32
At Peace	Secure Attachment	1.900	0.281	6.752	**<.001**
	Avoidant Attachment	1.887	0.349	5.408	**<.001**
	Anxious/Ambivalent	4.614	0.690	6.688	**<.001**
ACE > 0	Secure Attachment	−0.144	0.597	−0.241	.81
	Avoidant Attachment	−1.294	0.846	−1.530	.13
	Anxious/Ambivalent	−0.108	1.870	−0.058	.95
WCS	Secure Attachment	1.916	0.207	9.249	**<.001**
	Avoidant Attachment	2.231	0.255	8.740	**<.001**
	Anxious/Ambivalent	1.763	0.515	3.425	**.001**

Key: EIS = Existential Isolation Scale; EDS **=** Existential Distress Scale; DABBS = Death Anxiety Beliefs and Behaviors Scale; STS = Self-Transcendence Scale; Flourishing = Diener Flourishing Scale; Attachment = Adult Attachment Style; At Peace = item from the Quality of Life at the End-of-Life Measure (QUAL-E); ACE = Adverse Childhood Experiences; WCS = Watts Connectedness Scale. The at peace measure was centered at the median value of 4, and WCS was centered at the median value of 6.

We expected that three outcomes (existential isolation, existential distress, and explicit death anxiety) would reflect associations consistent with a lack of existential maturation, based on the conceptual model of existential maturation. Existential isolation was negatively associated with At Peace and Connectedness, and positively associated with male gender, ACE, and avoidant attachment (relative to secure attachment). Existential distress was negatively associated with At Peace and positively associated with male gender, ACE, and avoidant attachment (relative to secure attachment). For subjects with a secure attachment style, it was negatively associated with Connectedness. The explicit death anxiety (DABBS) outcome was negatively associated with at peace for subjects with a secure attachment style and positively associated with anxious/ambivalent attachment (relative to secure attachment).

We expected that two outcomes (self-transcendence, flourishing) would reflect associations consistent with existential maturation based on the conceptual model. Self-transcendence was negatively associated with avoidant adult attachment (relative to secure attachment) and positively associated with being at peace and connections (Table 7d). Flourishing was positively associated with being at peace and connections.

In our regression models, we coded ACE as “0 childhood-trauma events” or “greater than 0.”

Events.’ We also examined the models with ACE coded as 0–3 versus > 3. The conclusions were substantively similar, as shown in Supplemental Materials Table S3.

## Discussion

This large-scale panel study was conducted to further the development of a psychometric measure of existential maturation. We used 10 unique instruments, including the DABBS (a measurement of explicit death anxiety, which doubled as a mortality reminder) and a DTA word-completion task. DTA is a measure of how readily people associate with death words. This is understood to measure a person’s disposition to distance from, versus think unimpededly about, death matters. The assumption is that suppression of implicit death thoughts in response to a death reminder indicates nonconscious death anxiety. The study resulted in important findings, one of which was unexpected.

Our unexpected result relates to DTA. Previous studies (Arndt et al., 1997; Greenberg et al., 1994) have demonstrated that DTA scores decrease (indicating higher anxiety) immediately after a death reminder and subsequently rise (indicating anxiety subsiding) with distance from the reminder. This has been understood as an initial nonconscious defensive suppression of death thoughts, which we would expect to be most pronounced in subjects with low existential maturation. Accordingly, our initial hypothesis was that timing death reminders differently (DABBS before both DTA measures in the intervention group and subsequent to both DTA measures in the control group) would result in a significant difference in the outcomes. However, there is no evidence of DTA suppression in response to the mortality reminder in the present sample.

This result was unexpected and generative in our further thinking. It is possible that the lack of significant difference is related to the formatting of the prompts, as the visual structure of the DTA measure we employed was different from prior experiments. To prevent participants’ devices from auto-filling answers to word stem prompts (a hindrance not yet present in the studies conducted in the 1990s), it was necessary for each letter of the response to be typed separately. The letters were arranged horizontally on the computer screen (Figure 2) rather than on paper. It is unknown if the formatting may have obscured access to nonconscious responses, given the increased cognitive complexity of this task (Naidu et al., 2022). Alternatively, it is possible that the DABBS instrument was an ineffective induction of a mortality reminder. Future research could consider the possibility that DADDS (Krause et al., 2015; Lo et al., 2011; Shapiro et al., 2021), an alternative scale, could be a better mortality reminder (and measure of explicit death anxiety). Also, the current world is saturated with death reminders, such that potentially earlier mortality reminder inductions no longer produce detectable results. These possibilities are important to discern to achieve a measure of nonconscious responses to death salience.

We found remarkable outcomes in other measures. In the multivariate analyses, there were striking relationships between attachment style and variables considered as existential maturation outcomes, predictors, or interactions. These findings confirm studies that establish a connection between relationships and death anxiety (Verin et al., 2022). They also confirm the importance of including relationship-related items in a measure of existential maturation. Potentially, relationships are not only a buffer for death anxiety but a constituent part of the maturational process that is triggered by moments of realization that we are mortal.

Further, as expected, although there were significant positive correlations between EIS and EDS, and Flourishing and STS, the correlation was much lower between EIS and EDS (*r* = .29) than between Flourishing and STS (*r* = .65). This finding raises the possibility that EIS/EDS and Flourishing/STS are assessing related, but not identical, phenomena; i.e., that there are potentially independent positive and negative elements that contribute to existential maturation. This finding may also be considered consistent with the existential maturation model of recursive processes that can lead in positive or negative directions. As such, these measures may provide distinct and important features to our developing measure of existential maturation.

A notable feature of our study was our inquiry into participants’ experiences of deaths in the family. We asked that question last to avoid it being a contributor to the experimental mortality reminder. Correlations were not significant. Knowing that the impact of a death in the family is almost never insignificant, we consider it likely that our findings are consistent with the existential maturation model, in which a death experience can be traumatic and/or maturing. For our purpose of measurement, it also indicates that measuring the number of family deaths is not necessary in an optimal measure of existential maturation.

Finally, the correlations between the DABBS measure of explicit death anxiety and all of the other outcome variables, except existential distress (*r* = .42), were surprisingly low (r = .03, − .12, − .08). These findings indicate that explicit measures of death anxiety need to be supplemented by an implicit measure to adequately determine the role of death anxiety in the existential maturation process.

Overall, this study provided essential guidance to us in formulating the necessary components of a measure of existential maturation. We consider that such a measure will need at least the following components: a) an implicit measure of death anxiety, which would likely be best achieved using a subliminal induction and a free association lexical method to measure the impact; b) an explicit death anxiety measure, such as DADDS or DABBS; c) measures of essential relationships, including both those that formed attachment style and current relationships that aid a person’s processing; d) both positively and negatively covarying measures, such as those we used in the present study; and e) a method to measure oscillation in states of mind.

Limitations of this study include the aforementioned challenges with the formatting of the word-stem completion task, the unexpected possibility that the DABBS is not a sufficiently powerful mortality reminder in today’s population, recruitment under quota for some populations (Asian, Pacific Islander, American Indian/Alaskan Native, Hispanic), and restriction to English speakers and U.S. residents.

In conclusion, while this panel study provided unexpected results, our findings underscore the complexity and importance of measuring implicit death anxiety and offer fertile insights into possible pathways for future research, including an eventual measure of existential maturation.

## Supporting information

10.1017/S1478951525100497.sm001 Carr LaPorte et al. supplementary material Carr LaPorte et al. supplementary material

## Supplementary material

The supplementary material for this article can be found at https://doi.org/10.1017/S1478951525100497.

## References

[ref1] Arndt J, Greenberg J, Pyszczynski T, et al. (1997) Evidence for the active suppression of death-related ideation immediately after mortality salience: the effects of high cognitive load. *Journal of Personality and Social Psychology* 73, 5–18.9216076

[ref2] Brenner KO, Rosenberg LB, Cramer MA, et al. (2021) Exploring the Psychological aspects of palliative care: Lessons learned from an interdisciplinary seminar of experts. *Journal of Palliative Medicine* 24(9), 1274–1279. doi:10.1089/jpm.2021.0224 34469229

[ref3] Chochinov HM, Hack T, Hassard T, et al. (2005) Dignity therapy: a novel psychotherapeutic intervention for patients near the end of life. *Journal of Clinical Oncology* 23(24), 5520–5525. doi:10.1200/JCO.2005.08.391. 16110012

[ref4] Diener E, Wirtz D, Tov W, et al. (2010) New well-being measures: short scales to assess flourishing and positive and negative feelings. *Social Indicators Research* 97(2), 143–156. doi:10.1007/s11205-009-9493-y

[ref5] Emanuel L (2021) Psychodynamic contributions to palliative care patients and their family members. In Schwartz H (ed), *Applying Psychoanalysis to Medical Care*. New York, NY: Routledge, 111–124.

[ref6] Emanuel L (2023) Existential matters and quality of dying: A model of maturation processes. *Journal of Palliative Medicine* 26(12), 1604–1609. doi:10.1089/jpm.2023.0546 37824751

[ref7] Emanuel L and Brody S (2022) Psychodynamic therapy in the terminally ill. In Breitbart W and Chochinov H (eds), *Handbook of Psychiatry in Palliative Medicine*, 3rd. Oxford, England: Oxford University Press, 479–494.

[ref8] Emanuel L and Scandrett KG (2010) Decisions at the end of life: have we come of age? *BMC Medicine* 8, 57. doi:10.1186/1741-7015-8-57 20932275 PMC2964548

[ref9] Emanuel L, Solomon S, Fitchett G, et al. (2021) Fostering existential maturity to manage terror in a Pandemic. *Journal of Palliative Medicine* 24(2), 211–217. doi:10.1089/jpm.2020.0263 32552500 PMC7840299

[ref10] Felitti VJ, Anda RF, Nordenberg D, Williamson DF, Spitz AM, Edwards V and Marks JS (1998). Relationship of childhood abuse and household dysfunction to many of the leading causes of death in adults. The Adverse Childhood Experiences (ACE) Study. *American Journal of Preventive Medicine*, 14(4), 245–258. doi: 10.1016/s0749-3797(98)00017-8 9635069

[ref11] Florian V and Mikulincer M (1998) Terror management in childhood: does death conceptualization moderate the effects of mortality salience on acceptance of similar and different others? *Personality and Social Psychology Bulletin* 24(10), 1104–1112. doi:10.1177/01461672982410007

[ref12] Greenberg J, Arndt J, Simon L, et al. (1995) Testing alternative explanations for mortality reminder effects: Terror management, value accessibility, or worrisome thoughts? *European Journal of Social Psychology* 25(4), 311–321. doi:10.1002/ejsp.2420250406

[ref13] Greenberg J, Pyszczynski T, Solomon S, et al. (1994) Role of consciousness and accessibility of death-related thoughts in mortality salience effects. *Journal of Personality and Social Psychology* 67(4), 627–637. doi:10.1037//0022-3514.67.4.627 7965609

[ref14] Hayes J, Schimel J, Arndt J, et al. (2010) A theoretical and empirical review of the death-thought accessibility concept in terror management research. *Psychological Bulletin* 136(5), 699–739. doi:10.1037/a0020524. 20804234

[ref15] Hazan C and Shaver P (1987) Romantic love conceptualized as an attachment process. *Journal of Personality and Social Psychology* 52(3), 511–524. doi:10.1037//0022-3514.52.3.511 3572722

[ref16] Krause S, Rydall A, Hales S, et al. (2015) Initial validation of the death and dying distress scale for the assessment of death anxiety in patients with advanced cancer. *Journal of Pain and Symptom Management* 49(1), 126–134. doi:10.1016/j.jpainsymman.2014.04.012 24878066

[ref17] Krok-Schoen JLP-WA, Dailey PM and Krieger JL (2015) The conceptualization of self-identity among residents of Appalachia Ohio. *Journal of Appalachian Studies* 21(2), 229–246. doi:10.5406/jappastud.21.2.0229.

[ref18] Lo C, Hales S, Zimmermann C, et al. (2011) Measuring death-related anxiety in advanced cancer: Preliminary psychometrics of the death and dying distress scale. *Journal of Pediatric Hematology Oncology* 33(2), S140–145. doi:10.1097/MPH.0b013e318230e1fd 21952572

[ref19] Menzies RE, Sharpe L and Dar NI (2022) The development and validation of the death anxiety beliefs and behaviours scale. *British Journal of Clinical Psychology* 61(4), 1169–1187. doi:10.1111/bjc.12387 35938594 PMC9804815

[ref20] Naidu PA, Hine TJ and Glendon AI (2022) Methodological weakness of the death-word-fragment task: alternative implicit death anxiety measures. *Death Studies* 46(7), 1706–1715. doi:10.1080/07481187.2020.1846228 33186065

[ref21] Park YC and Pyszczynski T (2019) Reducing defensive responses to thoughts of death: Meditation, mindfulness, and Buddhism. *Journal of Personality and Social Psychology* 116(1), 101–118. doi:10.1037/pspp0000163 28836803

[ref22] Pinel EC, Long AE, Murdoch E, et al. (2017) A prisoner of one’s own mind: Identifying and understanding existential isolation. *Personality and Individual Differences* 105, 54–63 doi:10.1016/j.paid.2016.09.024.

[ref23] Pyszczynski T, Greenberg J and Solomon S (1999) A dual-process model of defense against conscious and unconscious death-related thoughts: an extension of terror management theory. *Psychological Review* 106(4), 835–845. doi:10.1037/0033-295x.106.4.835 10560330

[ref24] Pyszczynski T, Lockett M, Greenberg J, et al. (2021) Terror management theory and the COVID-19 Pandemic. *Journal of Humanistic Psychology* 61(2), 173–189. doi:10.1177/0022167820959488 38603072 PMC7498956

[ref25] Reed PG (1986) Developmental resources and depression in the elderly. *Nursing Research* 35(6), 368–374 doi:10.1097/00006199-198611000-00014. 3640355

[ref26] Russ V, Stopa L, Sivyer K, et al. (2024) The relationship between adult attachment and complicated grief: A systematic review. *Omega (Westport)* 89(4), 1293–1319. doi:10.1177/00302228221083110 35635029 PMC11423550

[ref27] Sagone E, Commodari E, Indiana ML, et al. (2023) Exploring the association between attachment style, psychological well-being, and relationship status in young adults and adults-a cross-sectional study. *European Journal of Investigation in Health Psychology and Education* 13(3), 525–539. doi:10.3390/ejihpe13030040 36975392 PMC10047625

[ref28] Schotanus-Dijkstra M, Ten Klooster PM, Drossaert CH, et al. (2016) Validation of the flourishing scale in a sample of people with suboptimal levels of mental well-being. *BMC Psychology* 4, 12. doi:10.1186/s40359-016-0116-5 26988345 PMC4794907

[ref29] Shapiro GK, Mah K, Li M, et al. (2021) Validation of the death and dying distress scale in patients with advanced cancer. *Psychooncology* 30(5), 716–727. doi:10.1002/pon.5620 33368836

[ref30] Smith A (1863) *DREAMTHORP: A Book Of Essays Written In The Country*. London: Strahan & Co.

[ref31] Steinhauser KE, Clipp EC, Bosworth HB, et al. (2004) Measuring quality of life at the end of life: validation of the QUAL-E. *Palliative and Supportive Care* 2(1), 3–14. doi:10.1017/s1478951504040027. 16594230

[ref32] Steinhauser KE, Voils CI, Clipp EC, et al. (2006) “Are you at peace?”: one item to probe spiritual concerns at the end of life. *Archives of Internal Medicine* 166(1), 101–105. doi:10.1001/archinte.166.1.101 16401817

[ref33] Steinman CT and Updegraff JA (2015) Delay and death-thought accessibility: a meta-analysis. *Personality and Social Psychology Bulletin* 41(12), 1682–1696. doi:10.1177/0146167215607843 26443599

[ref34] Vacchiano M, Politi E and Lueders A (2023) The COVID-19 pandemic as an existential threat: evidence on young people’s psychological vulnerability using a Multifaceted Threat Scale. *PLoS One* 18(10), e0292894. doi:10.1371/journal.pone.0292894 37824537 PMC10569596

[ref35] Verin RE, Menzies RE and Menzies RG (2022) OCD, death anxiety, and attachment: what’s love got to do with it? *Behavioural and Cognitive Psychotherapy* 50(2), 131–141. doi:10.1017/S135246582100045X 34852864

[ref36] Watson D, Clark LA and Tellegen A (1988) Development and validation of brief measures of positive and negative affect: the PANAS scales. *Journal of Personality and Social Psychology* 54(6), 1063–1070. doi:10.1037//0022-3514.54.6.1063 3397865

[ref37] Watts R, Kettner H, Geerts D, et al. (2022) The Watts Connectedness Scale: a new scale for measuring a sense of connectedness to self, others, and world. *Psychopharmacology (Berlin)* 239(11), 3461–3483. doi:10.1007/s00213-022-06187-5 35939083 PMC9358368

[ref38] Yalom ID (1980) *Existential Psychotherapy*. New York, NY: Basic Books.

