# Supplementary material for: Measuring American adults’ perceptions about human existence: A cross-sectional study
Source: Palliat Support Care. 2025 Aug 22;23:e143. doi: 10.1017/S1478951525100497 (PMC13166294; doi:10.1017/S1478951525100497)
Supplement: Carr LaPorte et al. supplementary material [file S1478951525100497sup001.docx]

**Supplemental Materials. Table 1. Demographics by Study group**

| Variable | Category | All | Group A | Group B | Group C | Group D | p |
| --- | --- | --- | --- | --- | --- | --- | --- |
| Age | | 49.1 (18.0) | 47.8 (17.7) | 48.8 (17.9) | 49.6 (18.5) | 50.1 (18.1) | .50 |
| Gender | Female | 509 (50.9) | 125 (50.2) | 126 (51.2) | 121 (50.4) | 137 (51.7) | .75 |
|  | Male | 483 (48.3) | 123 (49.4) | 119 (48.4) | 117 (48.8) | 124 (46.8) |  |
|  | Trans | 5 (0.5) | 1 (0.4) | 1 (0.4) | 2 (0.8) | 1 (0.4) |  |
|  | Other | 3 (0.3) | 0 (0) | 0 (0) | 0 (0) | 3 (1.1) |  |
| Education | < 8 yrs | 11 (1.1) | 3 (1.2) | 3 (1.2) | 2 (0.8) | 3 (1.1) | .72 |
|  | 8 – 11 yrs | 43 (4.3) | 5 (2.0) | 14 (5.7) | 9 (3.8) | 15 (5.7) |  |
|  | HS/GED | 268 (26.8) | 64 (25.7) | 66 (26.8) | 62 (25.8) | 76 (28.7) |  |
|  | Vocational | 61 (6.1) | 13 (5.2) | 17 (6.9) | 15 (6.3) | 16 (6.0) |  |
|  | Some college | 267 (26.7) | 80 (32.1) | 65 (26.4) | 56 (23.3) | 66 (24.9) |  |
|  | College | 262 (26.2) | 61 (24.5) | 61 (24.8) | 74 (30.8) | 66 (24.9) |  |
|  | Postgraduate | 88 (8.8) | 23 (9.2) | 20 (8.1) | 22 (9.2) | 23 (8.7) |  |
| Income | ≤ $9,999 | 118 (11.8) | 27 (10.8) | 27 (11.0) | 25 (10.4) | 39 (14.7) | .96 |
|  | $10,000 – $19,999 | 105 (10.5) | 15 (6.0) | 35 (14.2) | 27 (11.2) | 28 (10.6) |  |
|  | $20,000 – $34,999 | 196 (19.6) | 56 (22.5) | 48 (19.5) | 46 (19.2) | 46 (17.4) |  |
|  | $35,000 – $49,999 | 157 (15.7) | 48 (19.3) | 29 (11.8) | 40 (16.7) | 40 (15.1) |  |
|  | $50,000 – $74,999 | 184 (18.4) | 45 (18.1) | 48 (19.5) | 49 (20.4) | 42 (15.8) |  |
|  | $75,000 - $99,999 | 95 (9.5) | 22 (8.8) | 23 (9.3) | 25 (10.4) | 25 (9.4) |  |
|  | ≥ $100,000 | 113 (11.3) | 26 (10.4) | 28 (11.4) | 22 (9.2) | 37 (14.0) |  |
|  | Unknown | 32 (3.2) | 10 (4.0) | 8 (3.3) | 6 (2.5) | 8 (3.0) |  |
| Household | Very difficult | 167 (16.7) | 38 (15.3) | 45 (18.3) | 40 (16.7) | 44 (16.6) | .92 |
|  | Difficult | 184 (18.4) | 47 (18.9) | 38 (15.4) | 48 (20.0) | 51 (19.2) |  |
|  | Getting by | 325 (32.5) | 88 (35.3) | 81 (32.9) | 78 (32.5) | 78 (29.4) |  |
|  | Comfortably | 284 (28.4) | 66 (26.5) | 68 (27.6) | 65 (27.1) | 85 (32.1) |  |
|  | Unknown | 40 (4.0) | 10 (4.0) | 14 (5.7) | 9 (3.8) | 7 (2.6) |  |
| Ethnicity | Not Hispanic | 853 (85.3) | 211 (84.7) | 202 (82.7) | 210 (87.5) | 230 (86.8) | .28 |
|  | Hispanic | 142 (14.2) | 37 (14.9) | 43 (17.5) | 28 (11.7) | 34 (12.8) |  |
|  | Unknown | 5 (0.5) | 1 (0.4) | 1 (0.4) | 2 (0.8) | 1 (0.4) |  |
| Race | American Indian | 21 (2.1) | 6 (2.4) | 6 (2.4) | 4 (1.7) | 5 (1.9) | .68 |
|  | Asian | 34 (3.4) | 6 (2.4) | 4 (1.6) | 10 (4.2) | 14 (5.3) |  |
|  | Black | 125 (12.5) | 31 (12.4) | 33 (13.4) | 30 (12.5) | 31 (11.7) |  |
|  | Mixed | 35 (3.5) | 7 (2.8) | 9 (3.7) | 7 (2.9) | 12 (4.5) |  |
|  | Native Hawaiian / PI | 6 (0.6) | 2 (0.8) | 1 (0.4) | 3 (1.2) | 0 (0) |  |
|  | White | 749 (74.9) | 193 (77.5) | 181 (73.6) | 178 (74.2) | 197 (74.3) |  |
|  | Unknown | 30 (3.0) | 4 (1.6) | 12 (4.9) | 8 (3.3) | 6 (2.3) |  |
| Religion | Buddhist | 12 (1.2) | 5 (2) | 2 (0.8) | 3 (1.2) | 2 (0.8) | .32 |
|  | Christian | 590 (59) | 133 (53.4) | 157 (63.8) | 141 (58.8) | 159 (60) |  |
|  | Hindu | 4 (0.4) | 0 (0) | 0 (0) | 2 (0.8) | 2 (0.8) |  |
|  | Jewish | 40 (4) | 13 (5.2) | 6 (2.4) | 8 (3.3) | 13 (4.9) |  |
|  | Muslim | 22 (2.2) | 5 (2.0) | 3 (1.2) | 7 (2.9) | 7 (2.6) |  |
|  | Other religion | 51 (5.1) | 10 (4.0) | 14 (5.7) | 11 (4.6) | 16 (6) |  |
|  | Spiritual not religious | 66 (6.6) | 19 (7.6) | 17 (6.9) | 20 (8.3) | 10 (3.8) |  |
|  | No religion | 179 (17.9) | 55 (22.1) | 37 (15) | 37 (15.4) | 50 (18.9) |  |
|  | Prefer not to answer | 36 (3.6) | 9 (3.6) | 10 (4.1) | 11 (4.6) | 6 (2.3) |  |
| Rural | No | 771 (77.1) | 192 (77.1) | 188 (76.4) | 190 (79.2) | 201 (75.8) | .85 |
|  | Yes | 229 (22.9) | 57 (22.9) | 58 (23.6) | 50 (20.8) | 64 (24.2) |  |
| Rural Identity* | | 13.3 (8.6) | 13.9 (8.8) | 13.5 (8.7) | 12.4 (8.6) | 13.5 (8.5) | .25 |
| Death in immediate family | | 5.0 (5.0) | 5.0 (5.6) | 5.2 (5.1) | 5.2 (4.6) | 4.9 (4.6) | .33 |
| Adverse Childhood Experiences | | 2.1 (2.5) | 2.1 (2.5) | 1.9 (2.4) | 2.2 (2.5) | 2.2 (2.6) | .59 |

Key: *alpha = 0.92. The statistics were based on a scale without the city item. **Group A** - Group 1 with DTA-1 first; **Group B** - Group 1 with DTA-2 first; **Group C** - Group 2 with DTA-1 first; **Group D**- Group 2 with DTA-2 first.

**Supplemental Materials. Table 2. Descriptive statistics of outcomes and predictors by group.**

| Scale |  | All | Group A | Group B | Group C | Group D | p |
| --- | --- | --- | --- | --- | --- | --- | --- |
|  |  | **N (%)** | | | | |  |
| Adult Attachment | Avoidant | 348 (34.8) | 84 (33.7) | 75 (30.5) | 89 (37.1) | 100 (37.7) | .56 |
|  | Secure | 571 (57.1) | 142 (57) | 149 (60.6) | 132 (55) | 148 (55.8) |  |
|  | Anxious/Ambivalent | 81 (8.1) | 23 (9.2) | 22 (8.9) | 19 (7.9) | 17 (6.4) |  |
|  |  | **Mean (SD)** | | | | |  |
| Flourishing | total | 44.2 (9.2) | 44.0 (9.5) | 44.2 (9.0) | 44.0 (8.9) | 44.7 (9.3) | .81 |
| DABBS | total | 50.1 (15.2) | 51.2 (15.2) | 50.3 (14.6) | 48.3 (15.3) | 50.6 (15.6) | .19 |
| PANAS | posmood | 3 (0.9) | 2.9 (0.9) | 3 (0.9) | 2.9 (0.9) | 3 (0.9) | .23 |
|  | negmood | 1.9 (1) | 2 (1) | 1.9 (0.9) | 1.9 (1) | 1.8 (0.9) | .20 |
| WCS | Total | 5.8 (1.7) | 5.8 (1.7) | 5.7 (1.8) | 5.7 (1.8) | 5.9 (1.7) | .46 |
| STS | total | 45.7 (9.7) | 45.0 (10.0) | 45.8 (9.7) | 45.7 (9.7) | 46.4 (9.6) | .48 |
| EIS | total | 2.8 (1.0) | 2.9 (1.1) | 2.7 (1.0) | 2.7 (1.0) | 2.8 (1.0) | .30 |
| EDS | total | 12.9 (12.0) | 12.8 (12.4) | 13.2 (11.3) | 12.3 (12.0) | 13.3 (12.2) | .81 |
| At peace | score | 3.5 (1.3) | 3.4 (1.3) | 3.5 (1.3) | 3.5 (1.3) | 3.5 (1.3) | .81 |
| DTA 1 | score | 0.8 (0.8) | 1.0 (0.8) | 0.8 (0.8) | 0.8 (0.7) | 0.8 (0.8) | .001 |
| DTA 2 | score | 1.1 (0.8) | 1.2 (0.9) | 1.1 (0.9) | 1.2 (0.8) | 1.1 (0.8) | .45 |

**Key:** . Group A - Group 1 with DTA-1 first; Group B - Group 1 with DTA-2 first; Group C - Group 2 with DTA-1 first; Group D - Group 2 with DTA-2 first; Adult attachment=Adult Attachment Style; Flourishing=Diener Flourishing Scale; DABBS=Death Anxiety Beliefs and Behaviors Scale; PANAS=Positive and Negative Affect Scale-X; WCS=Watts Connectedness Scale; STS=Self-Transcendence Scale; EIS=Existential Isolation Scale; EDS**=**Existential Distress Scale; At Peace=item from the Quality of Life at the End-of-Life Measure (QUAL-E); DTA1=Death Thought Accessibility version 1; DTA2=Death Thought Accessibility version 2.

**Supplemental Materials. Table 3. Alternative regression model with different ACE dichotomization**

| **a. Outcome: Existential Isolation** | | | | | |
| --- | --- | --- | --- | --- | --- |
| **Predictor** | | **Estimate** | **Std Err** | ***t*** | ***p*** |
| Gender = Male | | 0.171 | 0.057 | 3.004 | **.003** |
| Attachment = Avoidant (Secure ref) | | 0.298 | 0.080 | 3.712 | **<.001** |
| Attachment = Anxious/Ambivalent (Secure ref) | | 0.244 | 0.139 | 1.761 | .08 |
| At Peace | Secure Attachment | -0.139 | 0.036 | -3.877 | **<.001** |
|  | Avoidant Attachment | -0.178 | 0.044 | -4.075 | **<.001** |
|  | Anxious/Ambivalent | -0.194 | 0.090 | -2.147 | **.03** |
| ACE > 3 | Secure Attachment | 0.332 | 0.104 | 3.207 | **.001** |
|  | Avoidant Attachment | 0.343 | 0.099 | 3.478 | **.001** |
|  | Anxious/Ambivalent | 0.154 | 0.244 | 0.630 | .53 |
| WCS | Secure Attachment | -0.194 | 0.026 | -7.479 | **<.001** |
|  | Avoidant Attachment | -0.119 | 0.032 | -3.724 | **<.001** |
|  | Anxious/Ambivalent | -0.097 | 0.065 | -1.492 | .14 |
| **b. Outcome: Existential Distress** | | | | | |
| **Predictor** | | **Estimate** | **Std Err** | **t** | ***p*** |
| Gender = Male | | 3.050 | 0.665 | 4.587 | **<.001** |
| Attachment = Avoidant (Secure ref) | | 3.054 | 0.942 | 3.243 | **.001** |
| Attachment = Anxious/Ambivalent (Secure ref) | | 4.847 | 1.624 | 2.985 | **.003** |
| At Peace | Secure Attachment | -2.740 | 0.421 | -6.515 | **<.001** |
|  | Avoidant Attachment | -3.645 | 0.513 | -7.109 | **<.001** |
|  | Anxious/Ambivalent | -1.020 | 1.058 | -0.965 | .33 |
| ACE > 3 | Secure Attachment | 6.104 | 1.213 | 5.031 | **<.001** |
|  | Avoidant Attachment | 4.776 | 1.156 | 4.131 | **<.001** |
|  | Anxious/Ambivalent | 3.743 | 2.862 | 1.308 | .19 |
| WCS | Secure Attachment | -1.106 | 0.304 | -3.641 | **<.001** |
|  | Avoidant Attachment | 0.113 | 0.374 | 0.302 | .76 |
|  | Anxious/Ambivalent | -0.470 | 0.761 | -0.617 | .54 |
| **c. Outcome: Explicit Death Anxiety (DABBS)** | | | | | |
| **Predictor** | | **Estimate** | **Std Err** | **t** | ***p*** |
| Gender = Male | | -0.092 | 0.948 | -0.097 | .92 |
| Attachment = Avoidant (Secure ref) | | 3.534 | 1.342 | 2.632 | **.01** |
| Attachment = Anxious/Ambivalent (Secure ref) | | 11.432 | 2.315 | 4.938 | **<.001** |
| At Peace | Secure Attachment | -3.115 | 0.600 | -5.196 | **<.001** |
|  | Avoidant Attachment | -1.216 | 0.731 | -1.664 | .10 |
|  | Anxious/Ambivalent | 2.414 | 1.508 | 1.601 | .11 |
| ACE > 3 | Secure Attachment | 4.570 | 1.730 | 2.642 | **.01** |
|  | Avoidant Attachment | 0.056 | 1.649 | 0.034 | .97 |
|  | Anxious/Ambivalent | -0.195 | 4.081 | -0.048 | .96 |
| WCS | Secure Attachment | -0.259 | 0.433 | -0.597 | .55 |
|  | Avoidant Attachment | 0.395 | 0.533 | 0.742 | .46 |
|  | Anxious/Ambivalent | 1.098 | 1.084 | 1.013 | .31 |
| **d. Outcome: Self-Transcendence** | | | | | |
| **Predictor** | | **Estimate** | **Std Err** | **t** | ***p*** |
| Gender = Male | | -0.295 | 0.383 | -0.770 | .44 |
| Attachment = Avoidant (Secure ref) | | -1.070 | 0.543 | -1.973 | **.05** |
| Attachment = Anxious/Ambivalent (Secure ref) | | -0.827 | 0.936 | -0.884 | .38 |
| At Peace | Secure Attachment | 2.573 | 0.242 | 10.619 | **<.001** |
|  | Avoidant Attachment | 2.889 | 0.295 | 9.778 | **<.001** |
|  | Anxious/Ambivalent | 2.998 | 0.609 | 4.920 | **<.001** |
| ACE > 3 | Secure Attachment | -0.106 | 0.699 | -0.151 | .88 |
|  | Avoidant Attachment | -1.277 | 0.666 | -1.916 | .06 |
|  | Anxious/Ambivalent | 0.974 | 1.649 | 0.590 | .56 |
| WCS | Secure Attachment | 2.792 | 0.175 | 15.947 | **<.001** |
|  | Avoidant Attachment | 2.967 | 0.215 | 13.777 | **<.001** |
|  | Anxious/Ambivalent | 2.827 | 0.438 | 6.451 | **<.001** |
| **e. Outcome: Flourishing** | | | | | |
| **Predictor** | | **Estimate** | **Std Err** | **t** | ***p*** |
| Gender = Male | | -0.218 | 0.453 | -0.481 | .63 |
| Attachment = Avoidant (Secure ref) | | -2.069 | 0.642 | -3.223 | **.001** |
| Attachment = Anxious/Ambivalent (Secure ref) | | 1.099 | 1.107 | 0.993 | .32 |
| At Peace | Secure Attachment | 1.838 | 0.287 | 6.408 | **<.001** |
|  | Avoidant Attachment | 1.874 | 0.350 | 5.359 | **<.001** |
|  | Anxious/Ambivalent | 4.968 | 0.721 | 6.890 | **<.001** |
| ACE > 3 | Secure Attachment | -0.858 | 0.827 | -1.037 | .30 |
|  | Avoidant Attachment | -0.674 | 0.788 | -0.854 | .39 |
|  | Anxious/Ambivalent | 3.085 | 1.952 | 1.581 | .11 |
| WCS | Secure Attachment | 1.929 | 0.207 | 9.310 | **<.001** |
|  | Avoidant Attachment | 2.219 | 0.255 | 8.708 | **<.001** |
|  | Anxious/Ambivalent | 1.652 | 0.519 | 3.185 | **.001** |

**Key:** EIS=Existential Isolation Scale; EDS**=**Existential Distress Scale; DABBS=Death Anxiety Beliefs and Behaviors Scale; STS=Self-Transcendence Scale; Flourishing=Diener Flourishing Scale; Attachment=Adult Attachment Style; At Peace=item from the Quality of Life at the End-of-Life Measure (QUAL-E); ACE=Adverse Childhood Experiences; WCS=Watts Connectedness Scale.
